# Comparison of the Pharmacological Profiles of Selective PDE4B and PDE4D Inhibitors in the Central Nervous System

**DOI:** 10.1038/srep40115

**Published:** 2017-01-05

**Authors:** Chong Zhang, Ying Xu, Han-Ting Zhang, Mark E. Gurney, James M. O’Donnell

**Affiliations:** 1 Department of Pharmacology and Toxicology, School of Medicine and Biomedical Sciences, State University of New York at Buffalo, Buffalo, NY, 14214, USA; 2 Department of Pharmaceutical Sciences, School of Pharmacy and Pharmaceutical Sciences, State University of New York at Buffalo, Buffalo, NY, 14214, USA; 3 Department of Behavioral Medicine & Psychiatry, West Virginia University, Morgantown, WV, 26505, USA; 4 Tetra Discovery Partners, Inc., Grand Rapids, MI 49503, USA.

## Abstract

Inhibition of cyclic AMP (cAMP)-specific phosphodiesterase 4 (PDE4) has been proposed as a potential treatment for a series of neuropsychological conditions such as depression, anxiety and memory loss. However, the specific involvement of each of the PDE4 subtypes (PDE4A, 4B and 4C) in different categories of behavior has yet to be elucidated. In the present study, we compared the possible pharmacological effects of PDE4B and PDE4D selective inhibitors, A-33 and D159687, in mediating neurological function in mice. Both compounds were equally potent in stimulating cAMP signaling in the mouse hippocampal cell line HT-22 leading to an increase in CREB phosphorylation. In contrast, A-33 and D159687 displayed distinct neuropharmacological effects in mouse behavioral tests. A-33 has an antidepressant-like profile as indicated by reduced immobility time in the forced swim and tail suspension tasks, as well as reduced latency to feed in the novelty suppressed feeding test. D159687, on the other hand, had a procognitive profile as it improved memory in the novel object recognition test but had no antidepressant or anxiolytic benefit. The present data suggests that inhibitors targeting specific subtypes of PDE4 may exhibit differential pharmacological effects and aid a more efficient pharmacotherapy towards neuropsychological conditions.

The cyclic AMP (cAMP)-specific phosphodiesterase 4 (PDE4) enzyme family is a critical component of signaling pathways involving multiple neurological diseases. This enzyme family consists of 4 subtypes (PDE4A-D) that are independently coded by different genes. Work over the past few decades has shown that pharmacological inhibition of the PDE4 enzymes has benefit in diverse animal models with regard to emotional and cognitive behaviors^1^,^2^,^3^,^4^. Administration of rolipram, a prototypical PDE4 inhibitor which does not distinguish between the PDE4 subtypes due to the absolute amino acid sequence conservation of the PDE4 active site, produces antidepressant-like effects in both animals and humans via stimulation of cAMP signaling in the brain^5^. On the other hand, chronic treatment of rats with various classes of antidepressants result in altered PDE4 enzyme expression in the brain, suggesting that the PDE4s are an integral component in their mechanisms of action^6^. Similarly, the anxiolytic-like effects of PDE4 enzyme inhibition was achieved by chronic administration of rolipram, which promotes neurogenesis in adult hippocampal neurons in mice when normal cell proliferation was impaired by the neurotoxin methylazoxymethanol acetate (MAM)^7^. The memory enhancing effects of inhibiting the PDE4 enzymes seems to adopt a more complex network of signaling^8^. Rolipram not only enhances memory function in healthy animals, but also reverses the memory deficits that are induced by the muscarinic acetylcholine receptor antagonist scopolamine, the NMDA receptor antagonist MK-801, the mitogen-activated protein kinase/ERK kinase (MEK) inhibitor U0126, or β-amyloid^9^,^10^,^11^,^12^.

Most centrally acting PDE4 inhibitors have accompanying tolerability issues such as emesis and diarrhea which keep them from being further studied in clinical trials or brought to market^13^. One possible explanation is the fact that current PDE4 inhibitors are mostly directed to the active site of the enzyme, which has absolute amino acid sequence conservation throughout all isoforms, and therefore do not demonstrate any subtype selectivity. The PDE4 enzyme family consists of 4 subtypes (PDE4A-D) that are independently coded by different genes. Of these, PDE4A, PDE4B and PDE4D are present in the brain, especially in the brain regions such as the prefrontal cortex, hippocampus, amygdala, and nucleus accumbens that regulate affect and the reward system^14^,^15^. The distribution pattern as well as subcellular compartmentalization of each subtype suggest that they may serve distinct roles in the central nervous system and provide a theoretical basis for the separation of therapeutic and adverse effects of PDE4 inhibitors^16^. For example, PDE4A or PDE4B deficient mice display anxiogenic-like behavior and may have impaired emotional memory^17^,^18^; PDE4D knockout mice exhibit delayed growth, improved memory and reduced sensitivity to rolipram for its antidepressant effects^19^; PDE4D mRNA decreases with age whereas PDE4B mRNA does not^20^. Very recently, the discovery of subtype selective, allosteric inhibitors of PDE4 has achieved much progress with the use of x-ray crystallography.

Each PDE4 gene encodes multiple variants through differential promotor utilization or splice variation that can be categorized into long, short and super short isoforms^21^. Long isoforms of PDE4 contain two upstream conserved regions termed upstream conserved region 1 (UCR1) and UCR2. Long forms of PDE4 dimerize through formation of a 4-helix bundle. UCR1 is a target of protein kinase A phosphorylation which increases cAMP hydrolysis 2–4 fold over basal levels depending upon the splice variant, while the open and closing of UCR2 over the active site regulates access by cAMP. Small molecules that are able to bind in the active site of PDE4 and at the same time interact with specific residues in UCR2 can close UCR2 over the active site and thereby inhibit the enzyme^22^. An example of such an allosteric inhibitor, D159687, that is selective for PDE4D was described by Burgin *et al*.^22^. D159687 acts as a partial inhibitor as it “closes” one catalytic site of the dimerized enzyme in a “trans” position, leaving the other catalytic site partially active. Similarly, there is a second, downstream C-terminal helix CR3 (Conserved Region 3) through which selective inhibition of PDE4B is achieved. A-33 is a compound discovered by Naganuma *et al*. that exhibits over 100 fold selectivity against PDE4B versus PDE4D^23^. A-33 favors a binding pose with CR3 especially at the single residue, Leucine 674 (compared to a Glutamine at this position for PDE4D), and “closes” the catalytic site of the enzyme^16^.

The present study compared the neuro-pharmacological effects of a PDE4B and a PDE4D selective inhibitor, A-33 and D159687, respectively, and revealed that they mediate differential neurological functions in naïve mice. First, we characterized the cAMP stimulating effects of A-33 and D159687 in a mouse hippocampal cell line HT-22. Second, their pharmacological effects *in vivo* were evaluated in mouse models that represent depressive, anxious behaviors and learning and memory capabilities, as well as emetic-like behaviors as a measurement of their side effects.

## Results

### cAMP accumulation in HT-22 cells is stimulated by A-33 and D159687

**Figure 1:**
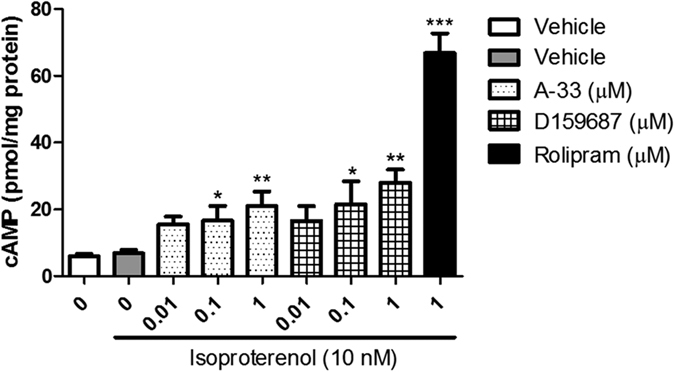
Accumulation of cAMP in the HT-22 cells treated with A-33 and D159687. The mouse hippocampal cell line HT-22 was treated with various concentrations of A-33 and D159687, and stimulated with isoproterenol (10 nM) for measurable cAMP signal. Results are expressed as mean ± SEM (n = 6). *P < 0.05, **P < 0.01 and ***P < 0.001 vs. vehicle treated isoproterenol-only group.

Both A-33 and D159687 increased cAMP accumulation in HT-22 cells in a dose-dependent manner as shown in Fig. 1. For A-33, 0.1 and 1 μM concentrations significantly increased the cAMP levels in the cells (F (3, 20) = 6.619, P = 0.0069); for D159687, the same concentrations significantly increased the cAMP accumulation (F (3, 20) = 6.165, P = 0.0061). The positive control drug rolipram (1 μM) induced a much larger increase in cAMP concentration in the HT-22 cells (P < 0.0001). Because the stimulatory effects for cAMP accumulation with both A-33 and D159687 were most obvious at 1 μM, this concentration was used for the subsequent time-dependent study on phosphorylation of CREB.

### Time- and concentration-dependent effects of A-33 and D159687 on phosphorylation of CREB in HT-22 cells

**Figure 2:**
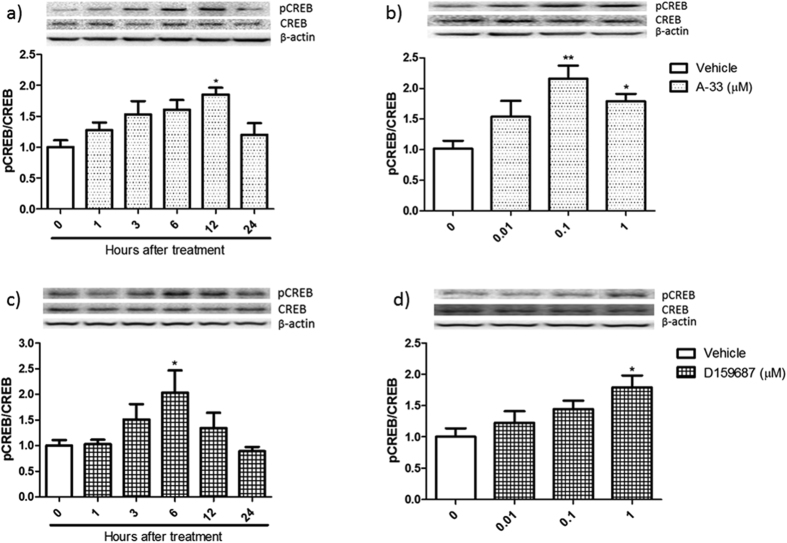
Time- and concentration-dependency of A-33 and D159687 in inducing phosphorylation of CREB. (**a**) HT-22 cells were treated with A-33 (1 μM) for 0, 1, 3, 6, 12 and 24 hours before sample collection. (**b**) HT-22 cells were treated with various concentrations of A-33 and incubated for 12 hours before sample collection. (**c**) HT-22 cells were treated with D159687 (1 μM) for 0, 1, 3, 6, 12 and 24 hours before sample collection. (**d**) HT-22 cells were treated with various concentrations of D159687 for 6 hours before sample collection. Results are expressed as mean ± SEM (n = 6). *P < 0.05 and **P < 0.01 vs. vehicle treated group.

To determine whether PDE4B and PDE4D inhibition indeed increased the phosphorylation of CREB as a downstream target of increased cAMP accumulation, we tested the optimal time after treatment and concentrations of the compounds that can lead to such effects (Fig. 2). Exposure of HT-22 cells to 1 μM A-33 induced a transient increase in phosphorylation of CREB which peaked at 12 hrs after treatment (Fig. 2a; F (3, 20) = 3.740, P = 0.0343). A lower concentration of A-33 (0.1 μM) induced higher levels of phosphorylation (Fig. 2b, F (3, 20) = 7.399, P = 0.0078). D159687 also induced a transient increase in CREB phosphorylation which peaked at 6 hrs after treatment (Fig. 2c, F (3, 20) = 3.731, P = 0.0425). CREB phosphorylation was optimal at 1 μM (Fig. 2d, F (3, 20) = 4.194, P = 0.0302).

### Measurement of depression-like behaviors in mice treated with A-33 and D159687

**Figure 3:**
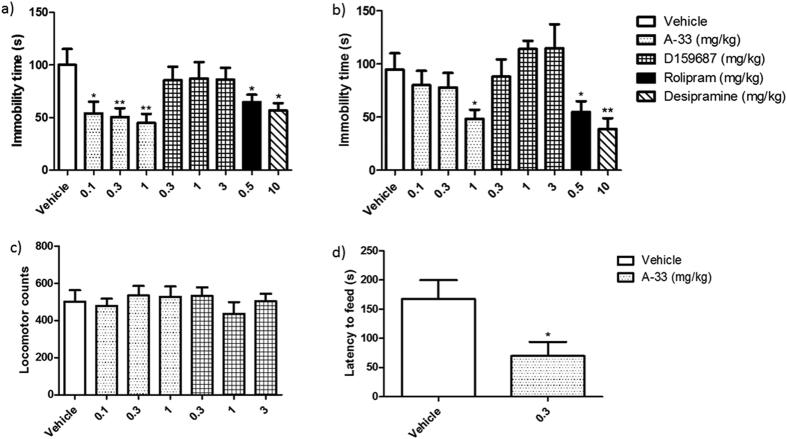
Evaluation of antidepressant-like effects of A-33 and D159687 on mice. (**a**) Mice were treated with vehicle, A-33, D159687, rolipram or desipramine 30 min before subjected to the FST. (**b**) Mice were treated with vehicle, A-33, D159687 rolipram or desipramine 30 min before subjected to the TST. (**c**) Mice were treated with various doses of A-33 and D159687 30 min before the locomotor activity test. (**d**) Mice were treated with vehicle or A-33 (0.3 mg/kg) for 7 days and subjected to the NSF 30 min after the last treatment. Results are expressed as mean ± SEM (n = 10–12). *P < 0.05 and **P < 0.01 vs. vehicle treated group.

The antidepressant-like effects of A-33 or D159687 were evaluated in the FST and TST after a single acute dose 30 min before testing (Fig. 3a,b). The PDE4B inhibitor A-33 exhibited strong antidepressant-like effects in both tests, with 0.1–1 mg/kg and 1 mg/kg being the effective doses in FST (Fig. 3a; F (3, 40) = 5.194, P = 0.0044) and TST (Fig. 3b; F (3, 40) = 3.182, P = 0.0471), respectively. The PDE4D inhibitor D159687, on the other hand, did not exhibit any antidepressant-like effects at the doses tested. Since the greater sensitivity in FST in some cases may represent a “false-positive” result^18^, we also performed the NSF test as a validation of the antidepressant-like effects of A-33 under a repeated-treatment regimen^24^. As shown in Fig. 3d, Mice treated with 0.3 mg/kg of A-33 for 7 days exhibited significantly reduced latency to feed (F (1, 18) = 1.600, P = 0.0245), suggesting that A-33 mimics the antidepressant like effects of the clinical antidepressants.

### Measurement of locomotor activity in mice treated with A-33 and D159687

The observed “antidepressant-like” effects of drugs can sometimes be interpreted as a general stimulation of the central nervous system of an individual. And in order to assess if the PDE4B and PDE4D inhibitors had any central stimulating or inhibitory effects on mice, we measured their locomotion counts 30 min after a single treatment of A-33 (0.1–1 mg/kg) or D159687 (0.3–3 mg/kg). As shown in Fig. 3c, neither compound affected locomotor activity, indicating that the antidepressant-like effects of A-33 were not due to central stimulation.

### Measurement of anxiety-like behaviors in mice treated with A-33 and D159687

**Figure 4:**
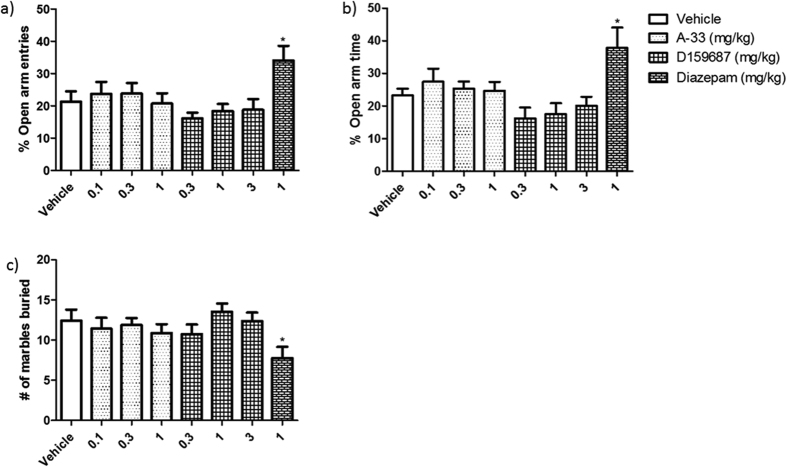
Evaluation of anxiolytic-like effects of A-33 and D159687 on mice. (**a** and **b**) Mice were treated with vehicle, A-33, D159687 or diazepam 30 min before subjected to the EPM. (**c**) Mice were treated with vehicle, A-33, D159687 or diazepam 30 min before subjected to the MBT. Results are expressed as mean ± SEM (n = 10–12). *P < 0.01 vs. vehicle treated group.

The potential anxiolytic effect of A-33 or D159687 were evaluated in two anxiety tests, EPM and MBT, after a single acute dose 30 min before testing. As shown in Fig. 4, neither compound exhibited any anxiolytic-like effects in % open arm entries (A-33 F (3, 40) = 0.2286, P = 0.8758; D159687 F (3, 40) = 0.6258, P = 0.6036), % open arm time (A-33 F (3, 40) = 0.3716, P = 0.7739; D159687 F (3, 40) = 1.253, P = 0.3076) in the EPM, or MBT (A-33 F (3, 40) = 0.3119, P = 0.8166; D159687 F (3, 40) = 0.9563, P = 0.4249). The positive control drug diazepam induced significant increases in the % open arm entries (P = 0.0341) and % open arm time (P = 0.0398) in the EPM, and decreased the number of marbles buried in the MBT (P = 0.0298).

### Measurement of memory- and cognition-related behaviors in mice treated with A-33 and D159687

**Figure 5:**
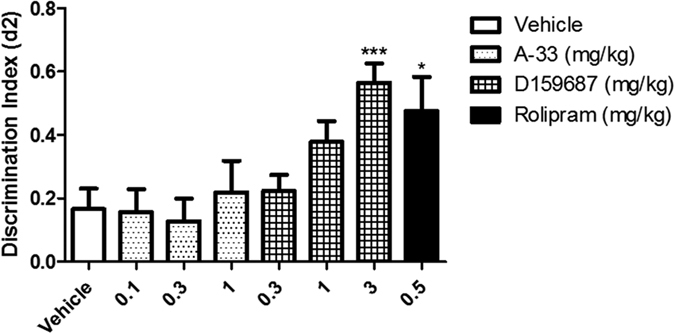
Evaluation of memory and cognitive enhancing effects of A-33 and D159687 on mice. Mice were treated with various doses of A-33, D159687 and rolipram (0.5 mg/kg) immediately after training on day 1, and subjected to the testing phase 24 hours afterwards. Results are expressed as mean ± SEM (n = 10–12). *P < 0.05 and ***P < 0.001 vs. vehicle treated group.

The NOR test assesses rodent memory under natural conditions, and does not require the pre-administration of memory-impairing agents^25^. The possible memory enhancing effects of A-33 and D159687 were evaluated in mice after a single acute dose given immediately after the T1 training session (Fig. 5). D159687 at 3 mg/kg significantly increased the discrimination ratio in T2 of the NOR test (F (3, 40) = 7.516, P = 0.0004). The benefit of D159687 was comparable to rolipram (P = 0.0139). A-33, in contrast, did not exhibited any memory enhancing effects at the doses tested.

### Evaluation of a pharmacological correlate of emesis in mice treated with A-33 and D159687

**Figure 6:**
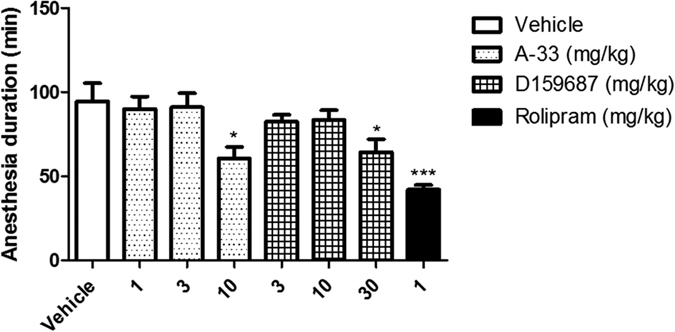
Evaluation of emetogenic potential of A-33 and D159687 in mice. Mice were anesthetized with Ketamine (80 mg/kg) and xylazine (10 mg/kg) 15 min before treatment with vehicle, A-33, D159687 or rolipram. Results are expressed as mean ± SEM (n = 8). *P < 0.05 and ***P < 0.001 vs. vehicle treated group.

Emesis is the major side effect of centrally acting PDE4 inhibitors that limits their tolerability^26^. Since rodents do not have an emetic reflex, the duration of ketamine/xylazine induced anesthesia has been introduced as a surrogate test for assessment of emetic potential in other species^26^. In the test, reduction of anesthesia duration by a PDE4 inhibitor correlates with emetic potency in other species^27^. We tested doses of A-33 and D159687 higher than the effective doses found in the previous behavioral tests so as to explore the potential window for tolerability. As shown in Fig. 6, A-33 and D159687 do not induce any shortening of anesthesia duration after treatment with ketamine and xylazine until 10 mg/kg (F (3, 28) = 3.455, P = 0.0252) and 30 mg/kg (F (3, 28) = 2.622, P = 0.0451), respectively. That was is 100 and 10 fold higher than the minimum effective doses in the behavioral tests for A-33 and D159687, respectively, giving a rather large therapeutic window for the compounds. The reduced emetic potential of the compounds as predicted by the anesthesia duration test is consistent with studies in shrews (D159687) and ferrets (A-33)^23^,^28^.

## Discussion

The absolute amino acid sequence conservation of the active site across all four PDE4 subtypes has made it difficult to develop subtype selective inhibitors^29^. Based on the work of Robichaud *et al*., PDE4D has been viewed as the emetic target of PDE4 inhibitors^30^. Therefore much effort has been made by medicinal chemists to develop allosteric inhibitors that are able to target specific subtypes which differ in their amino acid sequences in the regulatory domains. In the current study, while both PDE4B and PDE4D inhibitors induced accumulation of cAMP and increased phosphorylation of CREB in the HT-22 cells, their behavioral-pharmacological effects differ *in vivo*. PDE4B inhibition by A-33 exhibited a pure and strong anti-depressant like effect in the mouse FST and TST, with the FST being slightly more sensitive than the TST^18^. The antidepressant-like effect of A-33 was also confirmed by the NSF test under a repeated treatment regimen. PDE4D inhibition by D159687, on the other hand, did not induce any behavioral changes in mouse models of depression or anxiety, but improved cognition in the mouse NOR test, whereas as the PDE4B inhibitor had no procognitive benefit in healthy adult mice. The present data suggest that targeting these two subtypes of the PDE4 enzyme family might exert distinct functions in mediating behavior.

There are several lines of evidence supporting a critical role of PDE4B in the etiology of depression. First, PDE4B is mainly distributed in neural pathways that underlie reward and affect in mammals, and it was hinted that the antidepressant-like effects of rolipram may be through regulation of PDE4B^15^. Second, and probably the most intuitive evidence is that elevated PDE4B mRNA expression in the peripheral blood leukocytes is related to major depressive disorder (MDD) in humans and may be indicative of increased PDE4B expression in the brain^31^. Third, mice deficient in PDE4B showed decreases in baseline levels of monoamines and their metabolites within the striatum, and an “antidepressant-like” effect was indicated by a tendency of decreased immobility time in the FST^32^. The same knockout animals did not, however, exhibit changes in nociception or memory, while possible anxiogenic-like behaviors and impaired reversal learning in the Morris water maze were reported^18^,^33^. Fourth, an increasing number of studies suggest that chronic treatment with various antidepressants leads to decreased mRNA and protein levels of PDE4B^6^,^34^,^35^. Last but not least, pharmacological inhibition of PDE4B might benefit individuals with depression indirectly^36^. The Q31L mutation of the Disrupted-In-Schizophrenia-1 (DISC1) in mice is responsible for reduced activity of PDE4B, and the mutant mice exhibit increased sensitivity to other antidepressant treatment (GSK-3 inhibitor TDZD-8), suggesting that pharmacological inhibition of PDE4B, if not acting on its own, could at least prime the cellular environment so that other antidepressants become more effective^36^,^37^,^38^.

In the current study, the PDE4B selective inhibitor A-33 exhibited strong antidepressant-like effects in the FST and TST tests with slightly different sensitivity, which is not too surprising, as differential sensitivity has been reported for other antidepressant drugs^39^,^40^. Both tests suffer from common limitations such as an ambiguous definition of “loss of coping behavior”, which could be interpreted as an adaptation for “energy conservation” especially when chronic swim stressors are applied^41^. Therefore, the possible antidepressant-like effects of A-33 were also evaluated in mice using NSF under a chronic treatment regimen. Chronic, but not acute treatment of antidepressants is often required to induce a significant reduction in latency to food consumption, which reflects the therapeutic delay observed in the clinic. In the current study, a medium dose of A-33 (0.3 mg/kg) was able to mimic the effects of other antidepressants. These data suggest that the PDE4B selective inhibitor A-33 may serve as an effective novel antidepressant. Nevertheless, a role for PDE4B in other psychiatric diseases such as schizophrenia and bipolar disorder should not be ruled out, considering its rich expression in the striatum and close interaction with DISC1 as discussed above^32^.

The role of PDE4D in cognition has been well-characterized; first by studies of complete gene knockout, second by RNA-interference mediated knockdown, and finally by studies with other PDE4D selective inhibitors. Mice deficient in PDE4D showed memory enhancement in cognition tests which were comparable to the effects of chronic treatment of wild-type animals with rolipram^42^. The behavioral changes in the PDE4D knockout mice are attributed to increased hippocampal neurogenesis and enhanced cAMP/CREB signaling. Similarly, mice injected in the hippocampal CA1 region or the prefrontal cortex with lentiviral vectors for targeted downregulation of PDE4D4 and PDE4D5 also displayed enhanced cognition^42^,^43^. Furthermore, a novel PDE4D selective inhibitor, GEBR-7b, was shown to improve memory in both normal and APPswe/PS1dE9 mice, even though neither tau signaling nor Aβ levels were affected by the treatment^44^,^45^. This is not, however, contradictory with the idea of developing PDE4D selective inhibitors as a potential treatment for Alzheimer’s disease (AD), since more than 20% of individuals diagnosed with AD symptoms do not have amyloid plaque burden and are thus not responsive to antibody therapeutics^28^. NOR is a well-established cognition test for rodents, and is known for its ability to assess short- and long-term memory without stressful training or reinforcement conditions or a prolonged training schedule^25^. The improvement in cognition seen with D159687 is consistent with genetic manipulation of PDE4D expression^22^. Interestingly, in the current study, the PDE4D inhibitor does not affect behaviors related to depression or anxiety. Even though this is consistent with a previous report that an inhibitor of PDE4D does not exhibit significant antidepressant-like effects^45^, it is still somewhat contradictory with data from PDE4D deficient animals^19^. We speculate that the fundamental difference between a knockout animal in which compensatory mechanisms may have indirect impact on the behavioral phenotype^46^, and use of a selective inhibitor that only causes transient inhibition of the enzyme may be the major cause for such discrepancies^22^. PDE4D deficient mice also showed additional phenotypes unrelated to CNS function, such as impaired growth, reduced viability and reduced female fertility^47^.

Rolipram and the two approved PDE4 inhibitors, roflumilast and apremilast, are highly emetogenic in animals and humans after a single dose^13^,^48^. Tolerability improves with dose escalation (apremilast) or with chronic dosing^49^. The lack of tolerability is thought to be due to the inhibition of all PDE4 subtypes independent of their isoform which may be either a monomer or homo-dimer, and independent of their activation state due to protein kinase A (PKA) phosphorylation. Subtype selective inhibitors such as D159687 and A-33 show greatly enhanced tolerability^22^,^23^. D159687 targets the dimeric form of PDE4D and is sensitive to the PKA phosphorylation activation state of the enzyme through which it appears to achieve improved tolerability^22^. A-33 targets the monomeric form of PDE4B and achieves improved tolerability by sparing inhibition of PDE4D as PDE4D is thought to be the emetogenic target^16^,^23^,^30^.

The present study provides support for the development of subtype selective PDE4 inhibitors for CNS disorders. For the first time, our study compared two selective inhibitors of PDE4B and PDE4D, A-33 and D159687, and showed that both compounds are centrally active and differentially alter mouse behaviors. A-33 had antidepressant-like activity in mice, whereas D159687 exhibited clear cognitive enhancing properties, and both having enhanced tolerability compared to other PDE4 inhibitors. The present findings substantiate the rich therapeutic promise of subtype selective PDE4 inhibitors for treating a range of CNS disorders including neuropsychiatric diseases such as major depressive disorder and neurological disorders such as Alzheimer’s disease.

## Materials and Methods

### Cell culture

The HT-22 cells were a generous gift from Dr. David Schubert (The Salk Institute for Biological Studies, La Jolla, CA)^50^. Cell cultures were maintained in DMEM supplemented with 10% fetal bovine serum (FBS) and cultured at 37 °C in 5% CO_2_^51^. Cells were plated at 2 × 10^5^ cells/well on 6-well culture dishes and cultured overnight before treatment.

### Animals

Male ICR mice weighing 25–30 g were used (Harlan, Indianapolis, IN) for all of the experiments. Mice were kept in a temperature-controlled room under standard laboratory conditions, with a 12 h light/12 h dark cycle. All animals had free access to food and water, and were allowed at least 1 h of habituation before any experiments were carried out. All behavioral tests were performed during 9:30 am–16:30 pm and in accordance with the “NIH Guide for the Care and Use of Laboratory Animals” (revised 2011) and were approved by the Institutional Animal Care and Use Committee of State University of New York at Buffalo.

### Drugs and treatments

A-33 (CAS number 121604-72-6) and D159687 (CAS number 1155877-97-6) were prepared as described elsewhere^22^,^23^. For experiments on cell cultures, compounds were dissolved in 100% dimethyl sulfoxide (DMSO) at a concentration of 10 mM and then diluted into culture medium at a final DMSO concentration of 0.1%. For animal behavioral tests, the testing compounds were dissolved in vehicle composed of 5% DMSO, 5% Solutol and 90% saline; the positive control drugs desipramine and diazepam were dissolved in saline. In the behavioral tests, high doses of compounds that could potentially cause loss of subtype selectivity were avoided. Mice were given intraperitoneal injections in a volume of 10 ml/kg body weight.

### cAMP analyses

cAMP concentrations were measured using the enzyme-linked immunosorbent assay (ELISA). On the day of treatment, the attached HT-22 cells were washed with warm phosphate buffer saline (PBS) and then incubated for 10 min with PBS supplemented with various concentrations of A-33 or D159687 and further for 10 min with 10 nM isoproterenol in the system^52^. After incubation, PBS containing the drugs was removed and the plates were let dry roughly before 200 μl HCl (0.1 M) was added to each well to lyse the cells. Cell lysates were collected into Eppendorf tubes and centrifuged at 800 × g for 15 min. Supernatants were collected for immediate assay or stored frozen for assay later using the cAMP complete ELISA kit (Enzo Life Sciences, Farmingdale, NY) according to the assay protocol.

### Behavioral tests

#### Tail suspension test (TST)

The TST for depressive behavior in mice was carried out as described previously^53^. Mice were suspended from a stand arm 50 cm above the floor using masking tape for fixation, leaving the last 1 cm of tail exposed. The time each mouse remained immobile during the last 4 min of the 6 min test was recorded.

#### Forced swim test (FST)

The FST was carried out similarly to that described previously^54^. Briefly, mice were individually placed in glass cylinders (height: 25 cm; diameter: 10 cm; containing 10 cm depth of water at 24 ± 1 °C) for 6 min. A mouse was determined to be immobile when there were only small movements necessary to keep its head above water. The duration of immobility was recorded during the last 4 min of the 6-min testing period.

*Locomotor activity* was assessed using an open field chamber^55^. The floor of the chamber was divided into sixteen identical squares. Briefly, mice were individually placed in the center of the chamber and allowed to acclimatize for 15 min. The locomotor counts of mice were measured by counting the number of line crossings (with all four paws crossed the line) in the following 15 min.

#### Novelty suppressed feeding (NSF)

The NSF test was performed according to the methods reported previously with minor modifications^24^. Twenty-four hours after food deprivation, mice were individually placed in the corner of a Plexiglas chamber (L45 cm, W45 cm, H40 cm) containing a food pellet in the center. A stopwatch was immediately started afterwards to record the time lapse for the mouse to show interest to food. Interest to food was defined as the mouse approaching the food pellet with its forepaws and starting eating. Feeding latency was recorded during the 5-min test period.

#### Marble burying test (MBT)

Behavior in MBT was assessed as described previously with minor modifications^56^. Briefly, after a 1-h acclimation period in the testing room, mice were placed in a standard mouse cage containing 5 cm of bedding and 20 glass marbles evenly spaced in the cage. A wire-mesh lid is placed on the cage with food and water provided. The number of marbles buried at the end of a 30-min session was recorded. A marble was considered buried if at least 2/3 was beneath the surface of the bedding.

#### Elevated plus-maze test (EPM)

Behavior in the EPM was assessed as described previously^57^. The EPM apparatus consisted of two open arms (30 cm × 5 cm) and two closed arms (30 cm × 5 cm × 15 cm) that extended from a central platform (5 cm × 5 cm). The entire maze was elevated 50 cm above the floor. Each mouse was placed at the central platform and allowed to freely explore for 5 min. The total number of entries into, and the time spent in open and closed arms were recorded. An entry was defined as all four paws in an arm. The percentages of entries and time spent in open arms were calculated as number of open-arm entries and time spent in open arms divided by total arm entries and total time, respectively.

#### Novel object recognition (NOR)

The NOR test was performed in a Plexiglas open field box (L45 cm, W45 cm, H40 cm) as described elsewhere^58^. Briefly, the task procedure consists of three phases: habituation, training (T1) and testing (T2) phase. In the habituation phase (day 1), each animal is allowed to freely explore the apparatus for 5 min. Then the animal is returned to its home cage. During the training phase (day 2), a single animal is placed in the center of the open field containing two identical objects located on the diagonal of the field, and allowed to explore for 5 min. After a retention interval of 24 hr after T1, the animal was subjected to the testing phase for 5 min, during which one familiar object and one novel object were presented. The arena and objects were thoroughly wiped with 70% ethanol after each trial to avoid the presence of any olfactory trails. The animals were considered to be exploring the object when directing the nose to the object no more than 2 cm and/or touching/sniffing the object. Sitting on the object was not considered as exploration behavior. The times each animal spent exploring the objects were recorded. Time spent exploring the objects during T2 was indicated as ‘a’ and ‘b’, respectively. The discrimination ratio d2 was calculated as d2 = (b − a)/(b + a). Mice that did not reach a minimum exploration time of 20 s during T1 or T2 were excluded from analysis.

#### Ketamine/xylazine induced anesthesia

Alpha2 adrenergic receptor-mediated anesthesia was performed as described previously^27^. In brief, animals were anesthetized with xylazine (10 mg/kg) and ketamine (80 mg/kg) through i.p. injections. 15 min later, all animals were given a second and acute injection of various concentrations of testing compounds or rolipram as a positive control. Mice were placed in the supine position in V-shaped metal troughs during their anesthesia. The return of righting reflex was determined by when the animal no longer remained on its back and was able to return itself to the prone position three times within 30 s.

### Immuno-blot analyses

HT-22 cells were lysed with RIPA buffer containing protease and phosphatase inhibitors and centrifuged at 13,000 rpm for 30 min at 4 °C. Aliquots of supernatant containing 30 μg protein were separated using 10% SDS-PAGE as described previously^59^. Proteins from the gels were transferred to polyvinylidene difluoride membranes, blocked with blocking buffer (phosphate buffered saline containing 3% BSA and 0.1% sodium azide) and incubated with primary antibodies overnight at 4 °C (phospho-CREB at Ser 133 (abcam, Cambridge, MA), CREB (Santa Cruz Biotechnology, Dallas, Texas), β-actin (abcam, Cambridge, MA)). Labeled protein bands were detected using enhanced chemiluminescence (ECL) method and quantified using Quantity One 1-D Analysis Software.

### Statistical analyses

All data are presented as mean ± standard error of mean (SEM) and were analyzed by GraphPad Prism. For multiple comparisons, one-way analysis of variance (ANOVA) followed by Dunnet’s post-test was used for statistical evaluation. Student’s t test was used for comparisons between vehicle-treated and positive control drug treated groups. Statistical significance was set at P < 0.05.

## Additional Information

**How to cite this article**: Zhang, C. *et al*. Comparison of the Pharmacological Profiles of Selective PDE4B and PDE4D Inhibitors in the Central Nervous System. *Sci. Rep.* **7**, 40115; doi: 10.1038/srep40115 (2017).

**Publisher's note:** Springer Nature remains neutral with regard to jurisdictional claims in published maps and institutional affiliations.
